# A human monoclonal antibody based immunoenzymetric assay to measure Fel d 1 concentrations in cat hair and pelt allergenic extracts

**DOI:** 10.3389/falgy.2024.1417879

**Published:** 2024-07-15

**Authors:** Ronald L. Rabin, Derek Croote, Aaron Chen, Ekaterina Dobrovolskaia, Joyce Jia Wen Wong, Jessica Grossman, Robert G. Hamilton

**Affiliations:** ^1^ Center for Biologics Evaluation and Research (CBER), U.S. Food and Drug Administration (US-FDA), Silver Spring, MD, United States; ^2^ IgGenix Inc., South San Francisco, CA, United States; ^3^ Division of Allergy and Immunology, Department of Medicine, Johns Hopkins University School of Medicine, Baltimore, MD, United States

**Keywords:** allergy, allergen extract, cat, Fel d 1, allergen immunotherapy, IgE, IgG4, IEMA

## Abstract

In the United States, 19 allergen extracts of different specificities are standardized, which means that their potencies are determined in comparison to a US reference standard. For cat allergen extracts, potency is determined by measuring Fel d 1 content expressed in in Fel d 1 units, and with a unitage that correlates with skin test reactions (bioequivalent allergy units or BAU). Currently, Fel d 1 content is measured with a radial immunodiffusion (RID) assay that uses polyclonal sheep antisera to detect the allergenic protein by producing a white precipitin line in agar gel. However, the RID is considered cumbersome, and the polyclonal sera may qualitatively vary among animals and may recognize epitopes irrelevant to human allergic disease. In this report, we describe a quantitative two-site immunoenzymetric assay (IEMA) for Fel d 1 that uses immobilized capture and soluble biotin-labeled detection Fel d 1-specific human IgE monoclonal antibodies (mAb) that have been class-switched to IgG4. Together, they sandwich Fel d 1 molecules from extracts. Using purified natural Fel d 1 as a calibrator, the historically reported ∼4 micrograms Fel d 1/Fel d 1 unit assignment was directly measured in this mAb-based IEMA at 3.12 ± 0.24 micrograms of Fel d 1 per Fel d 1 unit. This IEMA appears to be equivalent to RID in the measurement of biological potencies of commercial cat hair and cat pelt extracts marketed in the United States.

## Introduction

Allergic responses to cat dander are highly prevalent in 10%–20% of the general population, and second only to house dust mite as the leading causes of perennial respiratory allergic disease (1). While 8 cat allergens have been identified (2), ∼95% of cat-allergic patients develop IgE antibody to the secretoglobin Fel d 1, which is considered the “major” cat allergen (3). Fel d 1 is a 35–38 kD tetramer (4) that is produced in cat salivary and skin sebaceous glands (5, 6). The discovery of Fel d 1 (referred to at the time as Cat allergen I) by Ohman (7) was soon followed by clinical trials demonstrating that subcutaneous allergen immunotherapy with cat dander allergen extracts containing Fel d 1 decreased clinical sensitivity to cat allergens (8).

Cat dander and pelt extracts are among the 19 allergen extracts of different specificities currently standardized for potency in the United States (9). Exploiting the near universal reactivity to Fel d 1, potency of cat extracts was initially defined in Fel d 1 “units,” according to its Fel d 1 concentration, with 1 unit being equivalent to ∼4 µg of Fel d 1 protein (10). Early testing of non-standardized cat extracts by CBER revealed that 6 of 10 manufactured lots had little or no detectable Fel d 1 present (internal CBER communication). Subsequently, CBER defined cat extract potencies according to skin test reactivity using the “intradermal dilution for 50 mm sum of erythema” (ID_50_EAL) test first as “allergen units” (AU) and then by bioequivalent allergy units (BAU) (11). Using ID_50_EAL testing, 5.0–9.9 Fel d 1 units/ml was considered equivalent to 5,000 BAU/ml, and 10–19.9 Fel d 1 units/ml was equivalent to 10,000 BAU/ml Based on these *in vivo* derived data, cat extracts are currently required to have 10–20 Fel d 1 units/ml, with an assigned potency of 10,000 BAU (11). The standardization process has improved potency consistency and the overall quality of cat extracts used for diagnostic skin testing and immunotherapy.

FDA CBER has established potency testing procedures and provided reagents to measure allergen extract potency. For standardization, Fel d 1 has been measured using a polyclonal sheep antisera that is distributed into an agarose gel for use in a classic radial immunodiffusion (RID) assay (11). However, the RID is cumbersome and the precipitation rings are difficult to read if they are not sharply defined. Moreover, the antigenic epitopes recognized by sheep IgG anti-Fel d 1 may be irrelevant to those recognized by human IgE antibody. By contrast, human monoclonal IgE antibodies specific for Fel d 1 from cat-allergic subjects are inherently relevant to human disease and are known to bind to 3 discrete linear epitopes on the Fel d 1 heterodimer (12). They thus provide relevant reagents for use in a new immunoenzymetric assay (IEMA, often referred to as a sandwich ELISA) which captures Fel d 1 molecules with an immobilized anti-Fel d 1 mAb against one epitope and detects with a second labeled anti-Fel d 1 mAb that binds to a second discrete Fel d 1 epitope. In this report, we describe an IEMA using Fel d 1 specific human IgG4 monoclonal antibodies that were cloned from human B cells producing IgE anti-Fel d 1.

## Methods

### Antibodies

IgE monoclonal antibodies (mAbs) were discovered by IgGenix from multiple cat-allergic individuals as previously described (13, 14). In brief, B cells were isolated, stained, and individually sorted into separate wells of 384-well plates. Cells were lysed, mRNA encoding the entirety of the transcriptome was reverse transcribed, and the resulting cDNA was amplified, barcoded using Nextera XT (Illumina, San Diego, CA), pooled, and sequenced on Illumina sequencing instruments. Sequencing reads were processed, separately for each single cell, to yield a gene expression counts matrix as well as full-length heavy and light chain sequences comprising the monoclonal antibody of each IgE-producing B cell.

DNA sequences encoding variable domains from IgE mAbs were cloned into human IgG4 expression vectors and milligram quantities of human IgG4 mAbs were expressed in Chinese hamster ovary cells and purified by one-step MabSelect SuRe™ PrismA protein A chromatography (Cytiva, Marlborough, MA). All IgG4 mAbs were expressed with the common S228P hinge-stabilizing mutation that prevents arm exchange (15)**.** Next, mAbs were screened for specificity to cat extract and, if positive, the cat allergens Fel d 1, Fel d 2, Fel d 4, and Fel d 7 on the ImmunoCAP system using IgG4 reagents.

Selection of the ideal anti-Fel d 1 mAb pairs that bound different non-competing Fel d 1 epitopes were identified by epitope binning using surface plasmon resonance (SPR) (Carterra, Salt Lake City, UT) with purified natural Fel d 1 (Indoor Biotechnologies, Charlottesville, VA) as previously described (14). Binning is a process by which a pair of antibodies are evaluated for binding to the same or a different epitope on an antigen. In this surface plasmon resonance-based analysis, one mAb, the ligand, is first immobilized on the sensor surface. Next, the antigen is injected and captured by the immobilized mAb. Last, a second mAb, the analyte, is injected. If there is an increase in resonance units upon injection of the second mAb, then the two mAbs are assigned to 2 separate epitope bins. If no increase is observed, the two mAbs are assigned to the same epitope bin. Two matched pairs of purified human IgG4 anti-Fel d 1 antibodies (IGX-0201 and IGX-0202 reactive to one Fel d 1 epitope and IGX-0203 and IGX-0204 reactive to another Fel d 1 epitope) were all biotinylated using water soluble NHS-biotin (Sigma Chemical Company, St. Louis, MO). A box titration analysis was performed to identify the purified capture and biotinylated detection mAb pair that optimally produced maximal working range with the lowest intra- and inter-assay variation in our prototype Fel d 1 two-site IEMA.

### Immunoenzymetric assay

We established an immunoenzymetric assay in which purified Fel d 1-specific human IgG4 mAb (clones IGX-0201 and IGX-0202) were individually immobilized on separate plastic microtiter plates [0.1 ml/well, 10 µg/ml in PBS, 18–24 h incubation at room temperature (RT)]. Following a PBS-0.05% Tween-20 buffer wash (3 times with 0.3 ml/well), plates were blocked with 0.3 ml/well of PBS-1% bovine serum albumin and incubated 1 h at RT. Following a repeat buffer wash, 0.1 ml/well of up to 11 serial dilutions of CBER's E8 reference cat hair allergen extract, purified nFel d 1 and dilutions of unknown commercial cat hair and pelt extracts were pipetted into their respective wells in antibody-coated microtiter plates and incubated for 1 h at RT. Plates were washed 3 times and biotin-labeled Fel d 1-specific human IgG4 mAbs (IGX-0203 and IGX-0204 in PBS-1%BSA, 1 µg/ml, 0.1 ml/well) were separately pipetted into different plates with the different capture antibodies. Following 1 h, plates were washed and streptavidin-horseradish peroxidase (EMD Millipore Corporation, Burlington, MA) diluted in PBS-1% BSA (1 µg/ml, 0.1 ml/well) was pipetted into all plates and incubated for 1 h at RT. After a final buffer wash (4 times, 0.3 ml/well) TMB (3,3’,5,5’ tetramethylbenzidine) chromogenic substrate was added to all wells (0.1 ml/well) and stopped with 1M H_3_PO_4_ when the top standard reached an optical density (OD) of 2.0 at 450 nm using a microtiter plate reader. Duplicate ODs were averaged and background well values that only received buffer (no cat extract) were subtracted. Data were analyzed with Graphpad Prism (Version 10) using non-4-parameter linear regression curve fit for sigmoidal curves and applying shared value constraints for upper and lower asymptote curves. Log2 EC50 values were compared to determine concentration of Fel d 1 (units/ml) of unknown, and to compute the Fel d 1 mass per unit (µg Fel d 1/unit Fel d 1).

Repeated box titration analyses were performed to determine the optimal parameters of the final IEMA format (e.g., ideal pair of purified capture and biotin-labeled detection mAbs, reagent concentrations and incubation conditions). We used both CBER's E8 cat hair reference allergenic extract and nFel d 1 as reference preparations to obtain calibration curves. Since the IEMA assay is designed to evaluate complex cat hair and pelt extracts for potency, it was required that the primary reference material contain the same complex protein profile of the test extracts be selected for routine use. The final parameters of optimized IEMA reagents are presented Table 1. For each plate, references and unknowns were measured in duplicate, with 11 serial 1:2 dilutions from columns 1–11. Column 12 Rows A-D and E-H were reserved for no-mAb coat controls, and buffer blank (no reagents), respectively. All incubations were done at RT and plates were washed between each step with PBS-0.05% Tween 20.

**Table 1:** Final Fel d 1 IEMA reagent source, concentrations, and incubation conditions.

Reagent	Source	Stock concentration	Dilution (final concentration)	Incubation
mAb IGX-0201-Purified (capture antibody)	IgGenix	5 mg/ml	1:500 (10 µg/ml)	Overnight-RT
Blocking solution		PBS-1% BSA		1 h-RT
mAb IGX-0204-Biotin (detection antibody biotin-conjugated)	IgGenix	1 mg/ml	1:1,000 (1 µg/ml)	1 h-RT
Avidin-horseradish peroxidase conjugate	EMD Millipore	1 mg/ml	1:4,000 (250 ng/ml)	1 h-RT
TMB (3,3’,5,5’ tetramethylbenzidine)	KPL			5 min-RT
E8 and E9 reference cat hair extracts	CBER	E8: 14.7 Fel d 1 u/ml E9: 14.9 Fel d 1 u/ml	0.0294 u/ml (1:500) 1:500	
Native purified Fel d 1 (nFeld1)	Indoor Biotech	30 µg/ml	60 ng/ml (1:500)	

## Results

Whole blood samples were collected from cat-allergic subjects and processed using the IgGenix SEQ SIFTER single-cell RNA-sequencing platform to yield full length heavy and light chain sequences of IgE mAbs (13). Variable domains from these IgE mAbs were expressed with an IgG4 constant region containing the commonly used S228P hinge-stabilizing mutation that prevents arm exchange and subsequently characterized for their specificity, affinity, and Fel d 1 epitope bin.

**Figure 1:**
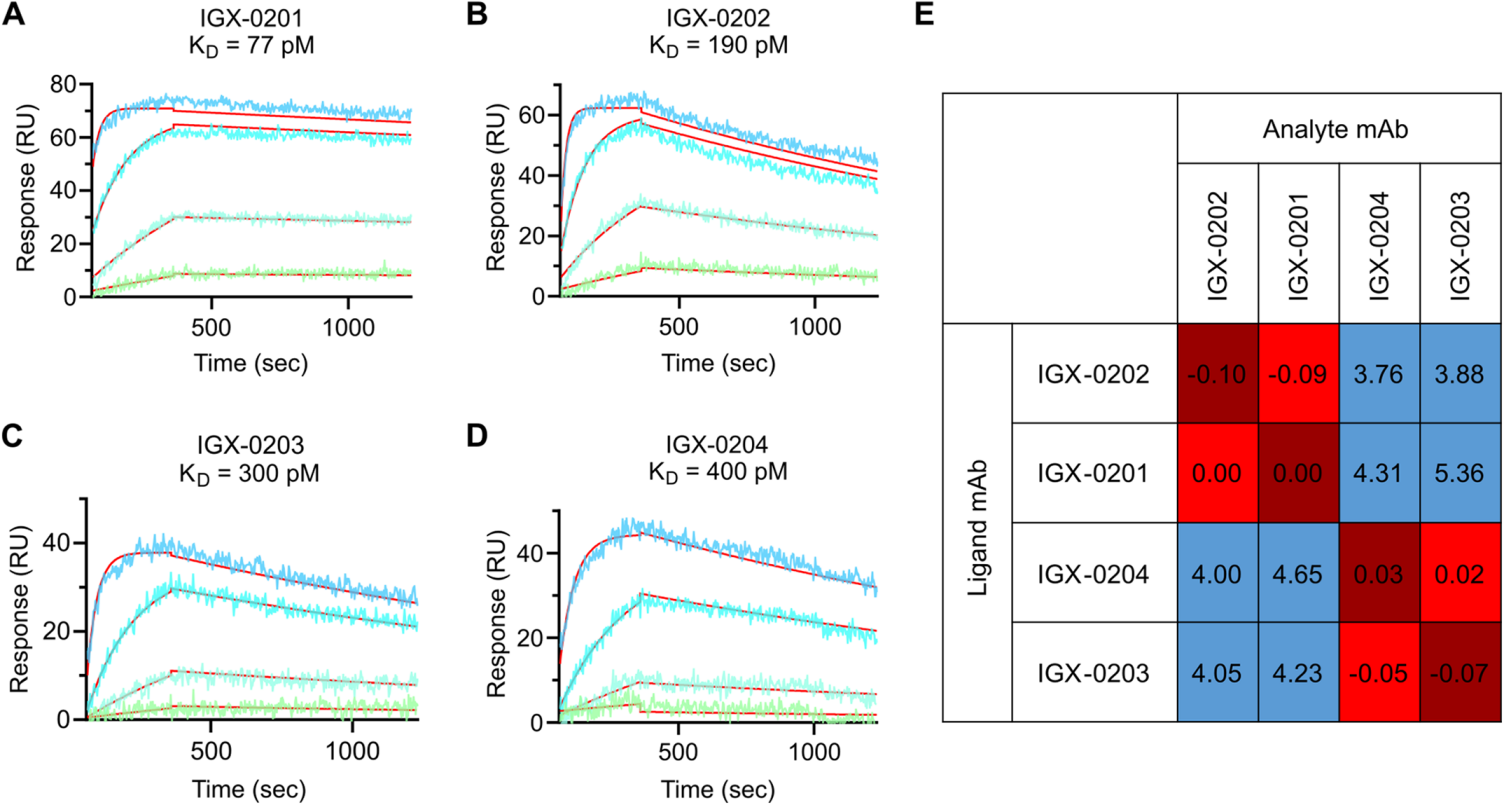
Affinity measurement and epitope binning of four human mAbs specific to Fel d 1. (**A**–**D**) Binding curves for each mAb against nFel d 1. Serial dilution of nFel d 1 used (blue to green): 20 nM, 4 nM, 0.8 nM, and 0.16 nM. pM = picomolar. (**E**) Pairwise epitope binning of nFel d 1. Blue = a pair of mAbs sandwiching nFel d 1; red = a ligand mAb blocks the binding of an analyte mAb to nFel d 1; dark red = a ligand mAb blocks itself as an analyte from binding nFel d 1 (see discussion of binning in the methods).

Figures 1A–D depicts high affinity binding to Fel d 1 intrinsic to the variable domains of four human IgE mAbs. All mAbs had sub-nanomolar affinity, specifically: 77 picomolar (pM), 190 pM, 300 pM, and 400 pM for IGX-0201 through IGX-0204, respectively. Pairwise competition using SPR demonstrated clear sandwiching and blocking interactions between these four mAbs that separated them into two discrete bins, one containing IGX-0201 and IGX-0202, and the other containing IGX-0203 and IGX-0204 (Figure 1E). Based on these bins, a box titration analysis was performed that identified IGX-0201 and IGX-0204 as the purified capture and biotinylated detection mAbs, respectively, that optimally produced maximal working range with the lowest intra- and inter-assay variation.

**Figure 2:**
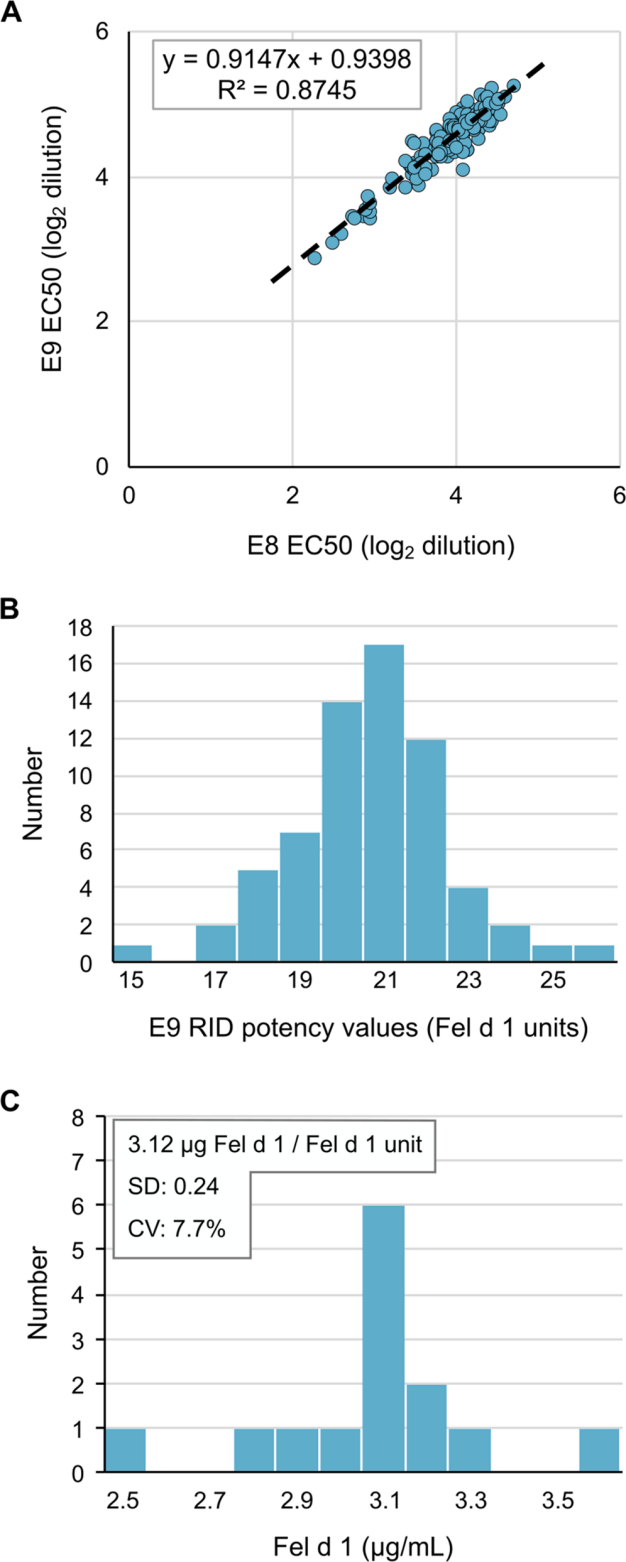
The Fel d 1 IEMA is reproducible and precise. (**A**) EC_50_ of two reference cat hair extracts reflects their relative potency, which was tightly distributed over 142 measurements. (**B**) Distribution of E9 extract potency values over 142 measurements by RID, using the E8 reference extract value of 14.7 Fel d 1 Units/ml. (**C**) IEMA determination of mass (ug) per unit of Fel d 1; *n* = 30.

After determining the optimal reagents and assay parameters for performing the IEMA, we repetitively measured Fel d 1 potency values in a new CBER reference cat extract E9 using E8 as the reference calibrator. Comparison of the concentration at 50% maximum response *(EC50)* from 142 separate IEMA plates (including those used to measure unknowns) showed a tight inter-assay correlation (*r*^2^ = 0.87, Figure 2A). Using the RID potency value for E8 of 14.7 Fel d 1 units/ml, the calculated potency of E9 was 21.3 Fel d 1 units/ml (SD 1.9, CV 8.9%, Figure 2B), which is substantially different from the RID potency value for E9 of 14.5 units/ml. We also repetitively measured nFel d 1 as a secondary standard to determine the mass/unit ratio which was computed to be 3.12 µg Fel d 1/Fel d 1 unit (SD 0.24, CV 7.7%, Figure 2C).

**Figure 3:**
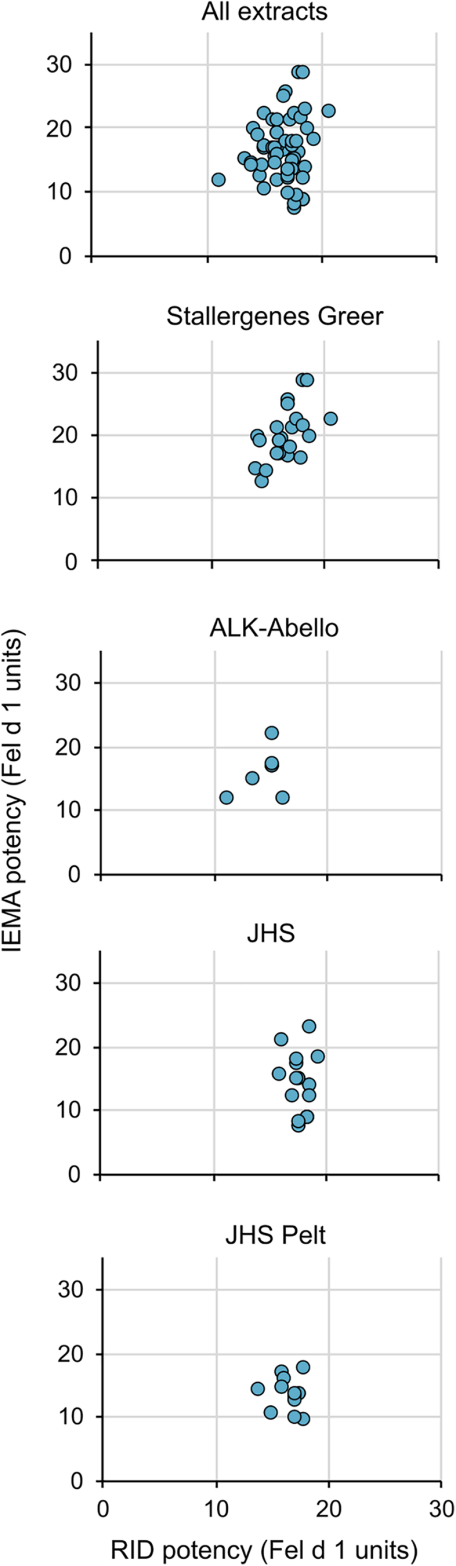
No correlation between potencies measured by RID and IEMA. The relationship between potency values as determined by RID and IEMA was not linear. All correlation coefficients in the figures were *r*^2 ^< 0.5. The top panel includes all extract data in a single graph while the lower panels show the trends broken down by company (panels 2 and 3) and source type (Hair—panel 4 vs. pelt—panel 5).

We then measured the potency values using the IEMA for 57 commercially-available cat extracts (45 cat hair, 12 cat pelt of different lots or batches) manufactured by three US extract manufacturers, Stallergenes-Greer, ALK-Abello, and Jubilant Hollister-Stier. Figure 3 (top) shows that while the relationship between potency values as determined by RID and IEMA was not linear (i.e., correlation was low; all *r*^2^ < 0.5), values were generally equivalent between the two assays. Figure 3 also shows that this was the case for cat hair extracts from each of the manufacturers as well as cat pelt extract, which is manufactured only by Jubilant Hollister-Stier.

## Discussion

Historically, allergen extracts and other biologics were first regulated by the US Hygienic Laboratory of the Public Health and Marine Hospital Service, the forerunner of the National Institutes of Health. In 1972, regulatory authority over biologics was transferred to the US FDA, and ultimately within the FDA, to CBER (11). CBER's authority to regulate extracts under 21 CFR has greatly improved their quality and safety by enforcing current Good Manufacturing Practices on their manufacturing processes, and by implementing standardization of selected allergen extracts. Standardized cat extracts were introduced into the US market in 1985 with potency defined by units of Fel d 1 (then referred to as “Cat allergen I”) in which 1 unit was considered equivalent to ∼4 µg Fel d 1 (10), and subsequently reported to be equivalent to 2.7 µg Fel d 1.

When introduced, ID_50_EAL testing in the skin of allergic individuals was considered a novel approach for determining potency of allergen extracts according to the magnitude of the observed *in vivo* allergic response (16). While not an *in vivo* test, the human mAb that are derived from human IgE mAbs discovered from cat-allergic individuals recognize epitopes that are inherently more relevant to the allergic response than is achievable with polyclonal animal antisera. The IEMA using highly avid mAbs reported here addresses the concerns of variable sheep antisera inter-lot potency and availability and the laborious nature of the RID. Its analytical sensitivity and inter-assay and inter-investigator reproducibility has shown it to be ideal as a potency monitoring assay for commercial extract standardization. We also believe that our IEMA using human IgG4 mAbs derived from cat-allergic individuals is more likely to track, ID50EAL potency than the current RID using antisera from hyperimmunized sheep. In this context, it is not surprising that potency measured using a pair of human mAbs that gives potency values that do not directly correlate with the potency measures obtained with polyclonal sheep antisera that likely recognizes more, and possibly different epitopes than the human IgE mAbs. In addition to recognizing two different allergenic epitopes, another obvious advantage of mAb use is their consistent, reproducible quality (specificity and affinity) over time that does not require laborious reference reagent replacement. Indeed, we (DC & RG) have previously demonstrated the potential of human mAbs discovered from peanut-allergic individuals to serve as highly specific reagents in a diagnostic application (17).

Revising the assay by which the potencies of cat allergen extracts are determined presents an opportunity to revisit the status of Fel d 1 as a “major allergen.” Lowenstein first used the term “major allergen” to refer to an allergen responsible for >50% of IgE binding in sera from a sensitized and objectively allergic population (18), although some investigators reserve that term for an allergen to which a large majority (as high as >90%) of allergic individuals react (19). In 2022, Roger and colleagues reported that 90% of 84 of cat allergic children and adults were reactive to Fel d 1 (20). In 2023, Ozuygur Ermis et al. reported that 84% (*n* = 304) of 361 cat-allergic Swedish adolescents and adults had IgE to Fel d 1 by ImmunoCAP, of whom 180 subjects were monosensitized. Additionally, 42% (*n* = 154) were sensitized to the lipocalins Fel d 4 or Fel d 7, while ∼12% were sensitized to Fel d 2 (albumin) (21). In 2023, Trifonova et al. used recombinant cat allergens and the basophil activation test to demonstrate that among 37 children and adolescents who were both sensitized and allergic (displayed objective symptoms) to cat allergens, 36 (97%) were reactive to Fel d 1, ∼70% were reactive to Fel d 4 and Fel d 7. Additionally, the median concentration to reach the plateau of basophil activation by Fel d 1 was 0.1 ng/ml, at least 10-fold lower than the other allergens tested (22). These studies support the view that Fel d 1 is the major cat allergen and an ideal protein to serve as an indicator of cat allergen extract potency.

Whether anti-Fel d 1 levels identify the majority of IgE antibodies responsible for the manifestation of cat allergy symptoms is less clear. All three clinical studies cited above found a substantial IgE antibody response to either lipocalins Fel d 4 and/or Fel d 7, and ∼60% of cat-allergic subjects had positive basophil reactivity to either or both recombinant lipocalins, albeit at higher concentrations than Fel d 1. Fel d 1 is also known as uteroglobin and is a member of the secretoglobin family (1). Fel d 1 is a tetramer formed by two identical heterodimer chains that are linked by a single disulfide bond (4). Using overlapping peptides, three IgE epitopes have been identified; two linear epitopes in chain 1 and one in chain 2 (12); an additional conformational epitope on chain 1 was identified with a mouse IgG mAb that effectively competed *in vitro* for human IgE antibody binding (23). Having multiple epitopes in combination with its property of dimerization (of two dimers) likely contributes to Fel d 1's property as a major allergen that can be readily cross-linked with low concentrations of IgE antibody. Moreover, supporting its status as a major allergen, subcutaneous administration of a pair of IgG4 mAbs in cat-allergic subjects, one against each chain of the Fel d 1 heterodimer, has been shown in to substantially decrease reactivity to nasal allergen challenge with cat extract (24).

In conclusion, we describe an IEMA for quantification of Fel d 1 using a pair of human IgG4 mAbs from cat allergic subjects specific for Fel d 1. This new assay appears to be equivalent to the current sheep polyclonal antibody-based RID in the measurement of potency of standardized cat allergen extracts. Review of recent literature supports continuing to use Fel d 1 as the major cat allergen by which potency of cat extracts is determined.

## Data Availability

The raw data supporting the conclusions of this article will be made available by the authors upon request, without undue reservation.
